# Chronic persistent Norovirus in the immune-compromised host

**DOI:** 10.1097/QCO.0000000000001152

**Published:** 2025-10-03

**Authors:** Anna Smielewska, Paraskevi Klotsas, Effrossyni Gkrania-Klotsas

**Affiliations:** a Clatterbridge Cancer Hospital, Alder Hey Children's Hospital and University of Liverpool Honorary Senior Lecturer Infection Biology & Microbiomes, LCL/CSSB, Liverpool University Hospital Foundation Trust, Royal Liverpool Site, Liverpool; b School of Medicine and Population Health, The University of Sheffield, Western Bank Villa, Western Bank, Sheffield; c Department of Infectious Diseases, Cambridge University Hospitals NHS Trust and Department of Medicine, University of Cambridge, Cambridge, UK

**Keywords:** common variable immunodeficiency, haematopoietic stem cell transplant, immune-compromised, norovirus, solid organ transplant

## Abstract

**Purpose of review:**

Norovirus is the most frequent cause of acute gastroenteritis globally, with increasing recognition of chronic infection among the immune-compromised. This review outlines the latest evidence on the pathogenesis, clinical features, and management of chronic norovirus infection in the immune-compromised host.

**Recent findings:**

Persistent norovirus shedding occurs in patients with compromised immunity, including those with congenital immunodeficiencies, recipients of solid organ and hematopoietic stem cell transplants, and individuals receiving chemotherapy or immunosuppressants. Chronic infection contributes to significant morbidity via prolonged gastrointestinal symptoms and malnutrition. The host cellular immune response, especially T cell function, is key to viral clearance. Limited therapeutic options, including ribavirin, nitazoxanide, and immunoglobulin, have shown mixed results. Investigational antivirals such as favipiravir are under early evaluation. Diagnosis remains complex due to histopathologic overlap with other enteropathies.

**Summary:**

Chronic norovirus infection in immunocompromised individuals remains an under-recognized and difficult-to-treat condition. Future progress requires development of evidence-based antiviral therapies, and effective vaccines to mitigate long-term morbidity.

## INTRODUCTION

Norovirus, a nonenveloped, single-stranded RNA virus in the Caliciviridae family, is a leading global cause of viral gastroenteritis [1]. In immunocompetent individuals, symptoms are typically acute and self-limiting. However, in immune-compromised hosts, norovirus can cause chronic infection with persistent viral shedding and an associated protracted and severe clinical disease characterized by significant electrolyte and nutrient losses as well as a need for total parenteral nutrition. Immunosuppression arising from primary immune deficiencies, organ transplantation, or cytotoxic therapies increases susceptibility to chronic infection [2]. Effective vaccination and treatment strategies are lacking. In this paper, we review the current knowledge on the pathogenesis and epidemiology of chronic norovirus infection of the immune-compromised host and focus on emerging prevention and treatment avenues. 

**Box 1:**
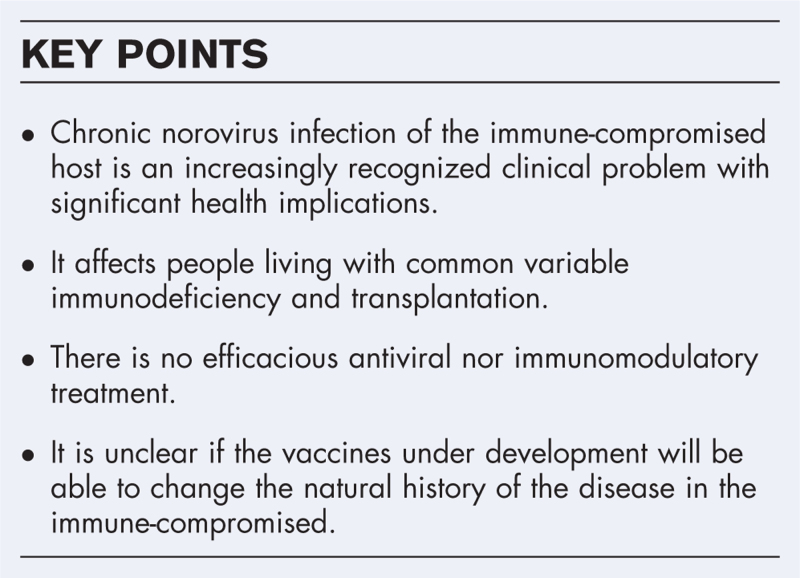
no caption available

## NOROVIRUS INFECTIONS IN HEALTHY PEOPLE

Norovirus infections are seasonal and highly contagious. In healthy people, norovirus associated acute symptomatic gastroenteritis is short-lived (48–72 h) and resolves spontaneously. Diagnosis is clinical. Laboratory diagnosis is possible via antigen-detection enzyme immunoassays but most commonly made by identification of the virus in stool by RT-PCR, which is also used for genotypic differentiation of viral strains. Viral shedding lasts up to three weeks [3]. Some hosts seem to be inherently partially or completely protected from becoming infected with specific norovirus genotypes [4], depending on the presence or absence of human histo-blood group antigens (HBGAs) on gut epithelial surfaces under the genetic control of the FUT2 (secretor), FUT3 (Lewis) and ABO(H) genes.

Healthy volunteer studies have shown that immunity to norovirus infections is also short-lived although at least a partial long term protection is suggested by the decrease of age-associated incidence [5]. In healthy individuals, norovirus clearance depends on both robust mucosal IgA responses and the generation of virus-specific memory B cells; these antibodies block viral attachment to gut epithelial cells and facilitate viral neutralization [5].

## NOROVIRUS INFECTIONS IN IMMUNE-COMPROMISED STATES

In contrast to the above, in immune-compromised hosts with primary or secondary immune deficiency, prolonged or even chronic infection can occur [6]. There is no formal definition of chronic norovirus infection so, for the purposes of this review, we define a chronic infection as the prolonged diarrhoeic illness associated with weight loss and malabsorption and that lasts more than thirty days during which the person continues to continuously shed the virus in their stool. Immune-compromised states that have been associated with chronic norovirus infection included solid organ transplants [7], allogeneic stem cell transplants [8] and patients with common variable immunodeficiency (CVID). Nosocomial outbreaks of norovirus among immune-compromised patients have also been well documented and pose substantial risk [9]. In immune-compromised hosts norovirus gastroenteritis can manifest with variable symptoms, from prolonged diarrhea to recurrent diarrhea, with or without vomiting, abdominal cramping and weight loss, leading to dehydration and nutritional deficiency [10]. Endoscopic biopsies may reveal villous atrophy, crypt hyperplasia, and increased intraepithelial lymphocytes, mimicking celiac disease or gastrointestinal graft-versus-host disease (GVHD) [11,12].

Immune control of norovirus is multifaceted, with T cells playing a central role [13]. CD8^+^ cytotoxic T lymphocytes help eliminate infected cells, while CD4^+^ T helper cells support B cell responses. In immune-compromised patients, a study in hematopoietic stem cell transplant (HSCT) recipients showed that viral clearance seems to coincide with T cell reconstitution [14^▪▪^]. Moreover, norovirus-specific immunoglobulin A (IgA) and IgG responses are often diminished in patients with hypogammaglobulinemia, underscoring the role of humoral immunity [15].

Chronic norovirus infections disproportionately affect HSCT and solid organ (SOT), especially renal and liver, recipients. In a case series of paediatric SOT and HSCT recipients from Texas [16], norovirus was the most common among other enteropathogens and led to recurrent diarrhea in 29% of the children. The annual incidence of norovirus associated diarrhoea in the SOT population has been estimated to be between 4% and 7% [17]. Limited studies estimate the risk of chronic (longer than two weeks) norovirus diarrhoea following acute norovirus infection among SOT recipients to be circa 30% [18]. Chronic norovirus diarrhea can become severe enough to cause a need for total parenteral nutrition and hospitalization [19^▪▪^,^20^]. Among haematopoietic stem cell transplant (HSCT) recipients, infection can result in significant prolonged healthcare utilization [21]. Additionally, in SOT recipients, norovirus infection can be associated with patient death and graft failures [22]. While some studies have investigated the secretor genotype or phenotype of the stem cell recipient, no information is yet available regarding the influence of the secretor status of the stem cell donors on the outcome of the norovirus infection in the recipient. Such studies on this important issue should be given priority.

Patients with CVID are particularly susceptible to norovirus infection – and to chronic, relapsing disease – because their underlying immunological defects affect B-cell differentiation in a way that leads to severely reduced serum and secretory immunoglobulins. Patients with primary immunodeficiencies such as CVID also exhibit a high prevalence of persistent norovirus shedding that can last from weeks to over a year [6].

In CVID, serum IgG is low by definition, and IgA is often profoundly reduced or absent. Loss of secretory IgA at the intestinal lumen permits norovirus to bind and infect enterocytes unimpeded. Moreover, many CVID patients exhibit defects in helper T-cell function and in the formation of germinal centers, further impairing affinity maturation of antiviral antibodies and the development of long-lived plasma cells capable of sustaining protective titers over time. The association between CVID and secretor status when it comes to norovirus risk has not been systematically reported upon.

## MANAGEMENT STRATEGIES

Current management of chronic norovirus disease in the immune-compromised host is largely supportive. Reduction in immunosuppressive therapy may promote immune recovery and viral clearance in transplant but must be weighed against the risk of graft rejection or GVHD exacerbation [23].

## ANTIVIRAL THERAPIES

Several antiviral agents have been tried with limited success.

## NITAZOXANIDE

Nitazoxanide is a thiazolide derivative, initially approved as an antiprotozoal agent. Evidence suggests it may function by modulating host cellular antiviral responses rather than directly inhibiting a specific viral enzyme [24]. Nitazoxanide is the antiviral candidate that has undergone the most extensive clinical evaluation for norovirus infection. An early Phase 2 randomized, double-blind, placebo-controlled trial in patients (aged ≥12 years) with acute gastroenteritis reported encouraging results including reduction of length of symptoms [25]. However, results from subsequent studies, particularly in immune-compromised populations with chronic infections, have not been encouraging [26]. A Phase 2 multicenter, prospective, randomized, double-blind study (2018–2021) evaluated nitazoxanide in 31 adult HSCT and SOT recipients with chronic norovirus infection (mostly GII genotype). This trial did not demonstrate a statistically significant benefit for nitazoxanide in shortening the median time to clinical resolution of symptoms or the time to the first negative viral load [27]. While nitazoxanide appeared safe and was well tolerated, any symptomatic improvement noted via patient-reported outcomes was considered transient.

Additionally, in vitro investigations using human intestinal enteroid (HIE) models, which more closely mimic human intestinal physiology, revealed that nitazoxanide did not exhibit high selective antiviral activity against any of the human norovirus strains tested [28].

## RIBAVIRIN

Ribavirin is a synthetic guanosine analogue with broad-spectrum antiviral activity. Its mechanisms of action are multifactorial, including the inhibition of inosine monophosphate dehydrogenase (IMPDH), leading to depletion of intracellular guanosine triphosphate (GTP) pools essential for viral RNA synthesis. Additionally, ribavirin triphosphate can be incorporated into viral RNA by RdRp, potentially causing mutations or acting as a chain terminator [29].

Some case reports describe successful treatment outcomes, such as the resolution of chronic norovirus infection in two CVID patients with oral ribavirin [6] Conversely, two case report detailed the development of ribavirin-resistant chronic norovirus infections in a CVID patient [30]. In vitro, ribavirin has demonstrated inhibitory activity against a human norovirus replicon [31], however the clinical success of ribavirin for norovirus is largely anecdotal and inconsistent; its use remains empirical with variable outcomes.

## FAVIPIRAVIR

Favipiravir is a broad-spectrum antiviral agent, a prodrug that is intracellularly phosphorylated to its active form, favipiravir-ribofuranosyl-5΄-triphosphate (favipiravir-RTP). This active metabolite is recognized by viral RNA-dependent RNA polymerase (RdRp) and incorporated into nascent viral RNA [32]. The primary mechanism of action is believed to be lethal mutagenesis and chain termination [33].

In animal (murine) and replicon studies, favipiravir has been shown to be effective in elimination of infection. In a clinical setting, a series of four immune-compromised patients (who also received nitazoxanide), favipiravir treatment was associated with an accumulation of viral mutations consistent with its proposed mechanism, coinciding with clinical improvement [34] However, viral clearance was not consistently achieved. Additionally, a case report documented the failure of favipiravir to effectively treat chronic norovirus infection in a kidney transplant recipient [35].

## IMMUNOGLOBULIN THERAPY

Intravenous immunoglobulin (IVIG) is an unreliable treatment for chronic norovirus, even among patients with CVID as most CVID patients who are also receiving regular IVIG continue to shed virus and have ongoing symptoms even in the face of adequate serum neutralizing levels [15]. Also, mucosal immunity (especially secretory IgA) is not replaced by IVIG. To replace the lack of mucosal immunity, oral immunoglobulin therapies have been used in patients with hypogammaglobulinemia but the use of these therapies remains unproven. Clinical reports describing the use of immunoglobulin enterally administered do not report any immediate or longer-term benefit [36^▪^].

## EMERGING THERAPIES AND VACCINE DEVELOPMENT

Experimental treatments targeting viral protease and host factors necessary for replication are under development.

There are a number of reasons why therapeutic and specifically direct-acting antiviral development has seen little progress. Virological factors include high genetic and antigen diversity that lead to difficulty in developing broadly effective direct-acting antivirals, rapid evolution and mutation, inherent to all RNA viruses, leading to rapidly emerging antiviral resistance as well as strain specific differences in clinical responses and host interactions [37] Basic research efforts have been hampered due to lack of simple norovirus cell culture models [38], leading to current HIE models that are limited by specific donors and difficulties in maintaining cultures in a research setting [39]. Animal models are mainly focused on murine norovirus which has significant limitations as a model for human infection [40]. Host responses are an additional challenge as they are strain specific and transient [41]. Additionally, susceptibility to infection with many common human norovirus strains is strongly influenced by host genetics, as mentioned above.

## EMERGING PROTEASE INHIBITORS

The norovirus 3C-like protease (3CLpro), is a viral protease essential for processing the large viral polyprotein (encoded by ORF1) into mature nonstructural proteins. The active site of 3CLpro is highly conserved across norovirus genogroups, making it an attractive target for broad-spectrum antiviral inhibitors [42], such as CDI-988 (Cocrystal Pharma). Preclinically, CDI-988 has demonstrated potent, broad-spectrum antiviral activity in vitro against multiple norovirus strains, and was assessed for safety and tolerability in a Phase I single centre study [43].

## EMERGING POL INHIBITORS

The viral RNA-dependent RNA polymerase (RdRp), or NS7, is another key enzyme essential for the transcription and replication of the norovirus RNA genome, making it a prime target. 2’-C-Methylcytidine (2CM-C) is a nucleoside analogue has demonstrated antiviral efficacy against norovirus in various experimental systems [44]. Other compounds like Suramin [45][46] and NF203 [47] have been identified as non-nucleoside inhibitors of norovirus RdRp.

## COMBINATION THERAPY

Despite the developments above, the range of direct-acting antivirals that are available for treatment of norovirus remains narrow. Combination therapy is likely to be a key strategy for achieving durable efficacy against norovirus, particularly in chronic infections or in immune-compromised hosts. For example, in vitro synergy has been reported for nitazoxanide combined with ribavirin against norovirus [48]. One CVID patient was treated sequentially with nitazoxanide, ribavirin, IFN-alpha, and finally immunoglobulins (which led to clearance), illustrating the use of multimodal approaches in difficult cases [15].

## ALTERNATIVE STRATEGIES: TARGETING THE HOST

HBGAs serve as crucial attachment factors or receptors for many human norovirus strains, and their expression is governed by host genetics, notably the FUT2 gene (secretor status) [49]. A potential therapeutic target could include the development of small molecule inhibitors of the FUT2 enzyme, thereby blocking viral attachment. However systemic inhibition of FUT2 could have unintended consequences, as FUT2 also plays roles in shaping the gut microbiota and other physiological processes [50^▪▪^].

Recent research using human intestinal enteroid cultures has revealed that bile is essential for the replication of certain human norovirus strains, such as GI.1 and GII.3 and this pathway is capable of exerting both proviral and antiviral effects during norovirus infections [51]. This suggests that modulating the bile acid signalling pathway could be a therapeutic strategy. However, there is still insufficient understanding of the complex anti and pro-viral effects that would necessitate a very nuanced approach. Modulating innate immune responses to viral infections, specifically interferon signalling may be another approach. Animal experiments using mice deficient in STAT1 or interferon receptors have shown that these defects make them particularly susceptible to norovirus infection [52]. Equally, it has been shown that interferon lambda (IFN-λ) (Type III IFN) can cure established MNV infection in mice, acting through IFN-λ receptors expressed on nonhematopoietic cells [53]. Therefore there is therapeutic potential for small molecule drugs that may boost the local production of IFN-λ [54].

Other modulation targets such as RIG-I (retinoic acid-inducible gene I) [55] and MDA5 (Melanoma Differentiation-Associated protein 5) have been explored as their overexpression can restrict both human norovirus and murine norovirus replication in vitro [56].

However, further understanding of the delicate modulation required to achieve this effect in these pathways is required.

## VACCINE DEVELOPMENT

There is currently no FDA-approved vaccine for norovirus but candidate vaccines are in various stages of clinical trials.

The most advanced seems to be Moderna's mRNA vaccine which is currently in phase 3 trials. The vaccine was initially placed on hold after a single incidence of Guillain-Barre syndrome during the phase 3 study. As of June 2025, the phase 3 trial is now fully enrolled [Moderna. Pipeline Overview. https://www.modernatx.com/pipeline. Accessed June 2025].

Vaxart has developed an oral tablet with an adenoviral vector expressing the norovirus GI.1 major capsid protein VP1, with Phase 1 trial data showing the induction of both mucosal and systemic immune responses [57].

Virus-like particle (VLP)-based vaccines and other candidate vaccines have shown promise in healthy children and adults [58]. Tak214 (Takeda) is a bivalent VLP vaccine targeting GI.1 and GII.4 genotypes. 2B trials have been completed and show high levels of immunogenicity [59], as well as hints of cross-genotype protection [60]. Immunogenicity appeared to wane over time. As of June 2025, Takeda has not announced the launching of a Phase 3 trial.

In addition to Takeda's work, several other norovirus vaccine candidates are under investigation.

Academic institutions and public agencies such as the National Institutes of Health (NIH) are also supporting several early-stage trials, including those assessing novel platforms such as nanoparticle delivery systems and intranasal vaccines. However, as of June 2025, these remain in preclinical or Phase 1 stages, with a current focus on safety and immunogenicity in healthy adult populations.

As described above, the high diversity of norovirus as well as the transient nature of the immune response as well the limited understanding of the immune correlate of protection continue to remain challenges.

So far, no norovirus vaccine trial has included immune-compromised hosts so immunogenicity and protective efficacy in this context is yet unclear. In the immune-compromised host, additional challenges for vaccine development will include antigenic diversity, rapid viral evolution [61^▪▪^] and impaired responses due to immunosuppression.

## CONCLUSION

Chronic norovirus infection in the immune-compromised host is an increasingly recognized clinical problem with significant health implications. There is no universally active vaccine or effective antiviral strategy. More and more people are becoming immune-compromised and live longer after transplantation or with a diagnosis of CVID. Existing and new immunomodulatory medications that have the potential to be associated with chronic norovirus are increasingly used. Progress will depend on the development of effective antiviral agents, and of vaccines suited to this vulnerable population.

## Acknowledgements

*None.*

### Financial support and sponsorship

*EGK is supported by the National Institute for Health and Care Research (NIHR) Cambridge Biomedical Research Centre (NIHR203312). The views expressed are those of the authors and not necessarily those of the NIHR or the Department of Health and Social Care.*

### Conflicts of interest

*There are no conflicts of interest.*

## REFERENCES AND RECOMMENDED READING

Papers of particular interest, published within the annual period of review, have been highlighted as:

▪ of special interest
▪▪ of outstanding interest
